# Public Health’s Inconvenient Truth: The Need to Create Partnerships With the Business Sector

**Published:** 2009-03-15

**Authors:** Elizabeth Majestic

**Affiliations:** Centers for Disease Control and Prevention

The corporate community and public health agencies [must] initiate and enhance joint efforts to strengthen health promotion and disease and injury prevention programs for employees and their communities.

*— IOM Report on the Future of Public Health in the 21st Century, 2003*

Why should public health agencies work with companies or trade associations that represent the business sector? Why should public health entities with altruistic goals work with companies that are primarily motivated by the desire for profit? The differences in mission, perspectives, and priorities create skepticism for public health officials about the motives of the private sector and the benefits of engaging in partnerships, even when the efforts might have substantial public health benefits. Because we face skyrocketing health care costs, a growing chronic disease burden, and shrinking resources for public health programs at local, state, and national levels, health professionals may reluctantly collaborate on specific projects with the business sector. These collaborations rarely establish the types of partnerships that promote the mutual exchange of ideas, resources, expertise, or access to specific populations, nor do they result in political advocacy that would benefit public health.

The interviews and articles in this theme issue of *Preventing Chronic Disease* (*PCD*) present a particular point of view in favor of working with the business sector that has not yet been fully embraced by the public health profession. Public health professionals have 2 compelling reasons to more actively and strategically seek partnerships with the business sector. The first reason is using the workplace to improve health. The second reason is based on changes in our society and their potential impact on public health partnerships.

## Using the Workplace to Improve the Health of Working Adults

Workplace health programs appeal to both the public health community and the business sector. Perhaps the most obvious reason is the opportunity to improve the health of millions of workers and their families. Because workers in America spend more than one-third of their day on the job, employers are in a unique position to protect their health and safety.

In the United States, the Centers for Disease Control and Prevention (CDC) has conducted research with employers to build the evidence needed to promote workplace health programs. This collaborative work with such organizations as the National Business Group on Health and the National Business Coalition on Health has produced guidelines and tool kits to help employers implement programs (www.cdc.gov/business).

Unhealthy workers are a major concern for the private sector. Escalating health care costs threaten the competitiveness of American business, even to the point of bankruptcy, and are a factor in the transfer of jobs overseas where it is cheaper to produce goods and services (1). Rising health care costs have contributed to the decline of American automakers. In 2007, General Motors, Ford, and Chrysler spent nearly $100 billion on health care for active workers, retirees, and their families, which added $1,500 to the cost of building each car. This clearly puts US automakers at a disadvantage with other countries where the government is responsible for workers' health care.

Healthy workers are good for business: they miss less work, are more productive, and have lower health care costs. In *Disease Prevention: A Job for Employers,* Lichiello and Harris note that more businesses are realizing that they need the expertise, tools, and resources offered by public health agencies to effectively promote worker health.

Employment-based health promotion offers employers, insurers, and government an opportunity to work together. . . . Recent research indicates that employers sorely need accurate, evidence-based information on health promotion activities from reliable, objective sources. They specifically want information on both the health impact and cost-effectiveness of health (2).

In fact, the private, for-profit sector is willing to pay our profession for its expertise, making the opportunity for partnership a win-win situation. Examples of these types of partnerships are the American Cancer Society's consulting program called Workplace Solutions (3), and CDC's collaboration with Cargill, Inc, on a research project that assessed worksite polices, programs, and practices. This last project was funded by Cargill and is described by Jason Lang and colleagues in this issue of *PCD* (4).

## The Changing Landscape

Public health professionals operate in a continually changing society. Our role includes anticipating these changes, identifying the shifting needs of the public, and preparing to meet these needs.

At times, rapid changes in political and social forces drive these changes. No one can underestimate the impact of the World Trade Center terrorist attacks on the US model of public health. The event challenged us to think about protecting the public's health in different ways. It showed us the importance of working with diverse agencies and organizations. And it expanded the range of partnerships in which we engaged — not just state and local health departments but advocacy groups, the business sector, and the public at large.

Substantial shifts in demographics and economic profiles also drive these changes. For example, because of the growing number of uninsured Americans and spiraling health care costs, health care has become a preeminent issue on state and federal public policy agendas. Reforming our health system will require a reexamination of the strengths, weaknesses, and boundaries of the sectors presently grappling with these issues: governmental public health, the health care industry, the private sector, and civil society institutions such as education agencies and national nonprofit organizations.

The following trends underscore the need to partner with the business sector: 1) the public's health has become big business; 2) there will be less money for public health programs; and 3) there is an increasing need for public health professionals but a shortage of workers.

Since 1960, when the National Health Expenditure Accounts (NHEA) began tracking medical expenditures by private insurance, public programs, and out-of-pocket spending, health care spending per capita has increased each year. In the past 3 decades, the total national spending on health care has more than doubled to 16% percent of the gross domestic product (GDP). In 2006 alone, national health expenditures grew 6.7% to $2.1 trillion, or $7,026 per person (5).

The Congressional Budget Office's (CBO's) 2008 projections in its Long-Term Outlook for Health Care Spending offered 2 possible scenarios, both of which reinforce the amount of money that could go into health care spending:

Under both scenarios total primary spending [for health] . . . would grow sharply in coming decades, CBO estimates, rising from its current level of 18% of GDP to more than 30% by 2082, the end of the 75-year period that CBO's long-term projections span.

### The public's health has become big business

Clearly health and health care are big business, and the private sector is responding enthusiastically. According to the Bureau of Labor Statistics:

As the largest industry in 2006, health care provided 14 million jobs with 7 of the 20 fastest growing occupations being in health care related fields. Health care will generate 3 million new wage and salary jobs between 2006 and 2016, more than any other industry (7).

As the business sector takes advantage of these new opportunities, health officials must recognize their related roles and responsibilities. In this issue of *PCD*, Eileen Salinsky notes the emergence of retail health clinics, demonstrating that health care is big business with public health responsibility:

As the nature of medical practice evolves, public health must continue to seek ways to harness the reach and creativity of new corporate stakeholders (8).

Working with the business sector can also be a responsibility taken on by national nongovernmental organizations. In his article about AARP's partnerships with the private sector, CEO Bill Novelli describes how his organization has formed a coalition of business associations and labor unions to ensure access to health care for all Americans (9). The Business Roundtable, the National Federation of Independent Business, and the Service Employees International Union have joined with AARP to form the *Divided We Fail* campaign to highlight the key issues of health care and financial security (www.aarp.org/aarp/presscenter/pressrelease/articles/DWF_Post_Election_Ads.html).

### There will be less money for public health programs

Public health revenues for programs at the national, state, and local levels will decline as a result of the economic downturn, growing budget deficits, and a shrinking workforce. As we experience the worst economic crisis since the Great Depression (10), tax revenues for local, state, and national governments will be reduced. Furthermore, while the US GDP is almost $14 trillion (11), in 2008, the US debt passed the $10 trillion mark (12), or more than half of our nation's GDP.

Similar budget troubles can also be seen at the state level. According to the Center on Budget Priorities:

At least 39 states faced or are facing shortfalls in their budgets for this and/or next year. Over half the states had already cut spending, used reserves, or raised revenues in order to adopt a balanced budget for the current fiscal year — which started July 1 in most states. New gaps have opened up in the budgets of at least 27 states plus the District of Columbia just 3 months after they struggled to close the largest budget shortfalls seen since the recession of 2001 (12).

The number of workers contributing to the economy will also decline in the foreseeable future. Today, there are 3.3 workers for every beneficiary. By 2030, the proportion of the US population aged 65 years or older will double to about 71 million, and the ratio of workers to beneficiaries is expected to decline even more, to 2.2 workers per beneficiary (13). (For more information, visit www.ssa.gov/OACT/ProgData/fundFAQ.html.)

The urgency of the current economic crisis requires that we forge partnerships with new stakeholders, especially the business sector. Without strong and effective programs, public health agencies run the risk of becoming irrelevant in addressing our nation's leading causes of death and disability. Public health agencies rarely have the resources needed to fully and comprehensively implement programs to address chronic diseases. With further reductions in funding, many public health programs will fail because they have insufficient resources to improve the public's health.

By combining the knowledge and expertise of public health with the resources of the business sector, health goals can be reached. In this issue of *PCD*, Easton, Davis and colleagues, and Hawkins and colleagues share experiences from the Steps Program, a part of CDC's Healthy Communities Program  (www.cdc.gov/HealthyCommunitiesProgram/), to demonstrate that collaborations with the private sector represent not only the possibilities but the realization of successful approaches to improving health (14-16).

### There is an increasing need for public health professionals but a shortage of workers

Chronic diseases such as asthma, cancer, diabetes, obesity, and heart disease affect the quality of life for 133 million Americans and are responsible for 7 of every 10 deaths in the United States, killing more than 1.7 million Americans every year. As the primary reason for escalating health care costs, chronic diseases account for more than 75 cents of every dollar we spend on health care in this country (17). Increases in foreclosures, joblessness, and people without health insurance will escalate the demands on public health programs. Advances in medical technology will extend the lives of the aging baby boomers and will place unparalleled demands on the nation's public health and health care systems.

As the need for public health increases, the number of public health workers is declining (17). The average number of public health workers varies greatly across the United States as measured by the 10 US Department of Health and Human Services regions, from 76 per 100,000 population in Region V to 200 per 100,000 population in Region 10.

Without concerted action, the situation will worsen. According to a 2004 joint report of the Council of State Governments, Association of State and Territorial Health Officials, and National Association of State Personnel Executives, "State governments could lose more than 30% of their workforce to retirement, private-sector employers, and alternative careers by 2006, and health agencies would be the hardest hit" (18). To address the growing labor shortage, Indiana University President Michael McRobbie discusses why he appointed a task force to assess the feasibility of establishing a school of public health and how his institution is pursuing partnerships with the private sector to improve the health of the people of Indiana (19).

## Building the Public Health Community's Capacity for Collaborating With the Business Sector

Creating and managing these partnerships requires that health officials develop a different set of competencies and capabilities. In his interview, CDC's former general counsel Gene Matthews encourages health officials to move beyond the absolutist approach concerning the business sector and find sophisticated partnership models that preserve institutional integrity while improving public health impact (20).

Although our profession should be seeking new and creative ways of responding to these trends by building partnerships with businesses, these collaborations must begin carefully. To be sure, some of the barriers to partnerships, such as those with the tobacco industry, have been erected by the public health community for legitimate reasons. Other partnerships, such as working with food companies that are owned by the tobacco industry to address the obesity epidemic, have in hindsight been questionable. In an interview in this issue of *PCD,* Michael Eriksen, Director of the Institute of Public Health at Georgia State University, shares his views about whether partnerships with the tobacco industry are possible and what lessons can be learned from the tobacco control experience that might be relevant for addressing the obesity epidemic (21).

Effective partnerships include a contractual agreement between a public health entity and 1 or more for-profit business organizations or trade associations. Through such agreements, the skills and assets of the public health entity and the business organization are shared to improve public health. In addition to sharing resources, each party also shares in the risks and rewards that result from the partnership.

To learn from our successes and share our failures, partnerships between public health and business must be systematically evaluated and reported in the scientific literature. In this issue's Tools and Techniques section, Fran Butterfoss provides useful information about evaluating these partnerships and offers a window into the diverse views held by the public and business sectors concerning evaluation and how to measure success (22).

In his article, Bob McKinnon suggests that the public and business sectors confront each other with antagonism and suspicion because they fail to understand and accept their differences. McKinnon explains that many problems stem from lack of a common language, a problem that could be ameliorated if both sectors worked to understand and address these communication barriers (23).

The Prevention Research Centers, as Sharon McDonnell and colleagues describe, are building public health research capacity through partnerships with the business sector. Several centers have implemented innovative projects, which exemplify not only the creative potential but the recognition of winning approaches to improving public health (24).

To help its employees develop effective partnerships, CDC developed an agency policy that contains criteria for assessing the appropriateness of a partnership with an outside agency, organization, business, or trade association. To implement the agency's policy, CDC's National Center for Chronic Disease Prevention and Health Promotion (NCCDPHP) developed additional guidance for its divisions (Appendix). The purpose of this guidance is to 1) provide specific criteria against which all public-private partnerships will be measured and approved to ensure the best interest of NCCDPHP; 2) provide a consistent, fair, and transparent review process for all public-private initiatives; and 3) provide a high level of confidence that the public's interests are fully assured through provisions in agreements and contracts for ongoing monitoring and oversight of the agreement.

As Goldsmith and Eggers observe:

Government agencies, bureaus, divisions, and offices are becoming less important as direct service providers, but more important as generators of public value within the web of multiorganizational, multigovernmental, and multisectoral relationships that increasingly characterize modern government (25).

For this reason and other reasons outlined above, public health needs to collaborate with the business sector to protect and improve the health, economic prosperity, and quality of life of the American people. Although public health and the business sector each bear a responsibility to assure the health of our nation, only by exercising those responsibilities together will we be able to contribute fully to that goal.

## Appendix

### National Center for Chronic Disease Prevention and Health Promotion Guidance for Establishing a Partnership Agreement With the Private Sector: Memorandums of Agreement; Memorandums of Understanding; CDC Foundation Projects

The brochure described in this article is available for download as a Microsoft Word document. You must have Microsoft Word to use this file.

http://www.cdc.gov/pcd/issues/2009/apr/images/08_0236_01.doc (DOC 43k)
